# An RM-ODP method to model PBL digital twins for supporting closed-loop supervision^☆^

**DOI:** 10.1016/j.mex.2026.104118

**Published:** 2026-08-27

**Authors:** Afonso Cesar Lelis Brandão, Reginaldo Arakaki, Leandro Augusto da Silva

**Affiliations:** a Instituto de Tecnologia e Liderança (Inteli), São Paulo, Brazil; b Universidade Presbiteriana Mackenzie, São Paulo, Brazil; c Universidade de São Paulo (USP), São Paulo, Brazil

**Keywords:** Digital twin, Project-based learning, Complex event processing, Learning analytics, Repository evidence, Data contracts, Process observability

## Abstract

Project-Based Learning (PBL) in software engineering produces heterogeneous evidence across repositories, task boards, classroom activities, and assessments, making timely supervision difficult. This paper presents a two-stage method to model PBL digital twins using the five RM-ODP viewpoints, aligned with ISO/IEC 10746 and ISO/IEC/IEEE 42010. Stage A models the as-is PBL ecosystem using SADT/IDEF0 and identifies native event points and requirement gaps. Stage B introduces a Digital Twin layer that consumes these events, applies data contracts, correlates patterns through Complex Event Processing (CEP), and delivers feedback within the active sprint window without replacing existing processes. The method structures goals and governance (enterprise viewpoint), semantic contracts and traceability (information viewpoint), event services and CEP rules (computational viewpoint), distributed pipelines and immutable snapshots (engineering viewpoint), and implementation choices (technology viewpoint). An institutional case instantiation demonstrates end-to-end applicability from process modeling to operational supervision signals. The result is a reusable procedure for supporting closed-loop supervision, auditable evidence integration, and longitudinal learning analytics in diverse PBL environments. In the current research cycle, Stage A is fully instantiated and Stage B is operational for repository-based evidence; correlation is executed as batch replay over sprint windows rather than as a streaming engine, so that the supervision-signal loop is demonstrated while the intervention-response loop that records how signals change pedagogical practice remains planned. The validation section quantifies this maturity and explicitly separates implemented, partially implemented, and planned capabilities.

## Method highlights

• Two-stage method that models PBL ecosystems and evolves them into Digital Twin supervision architectures.
• Integration of RM-ODP viewpoints with event-driven observability and Complex Event Processing.
• Reusable architecture for continuous evidence collection and sprint-window supervision signals in PBL environments.

## Graphical abstract

**Figure:**
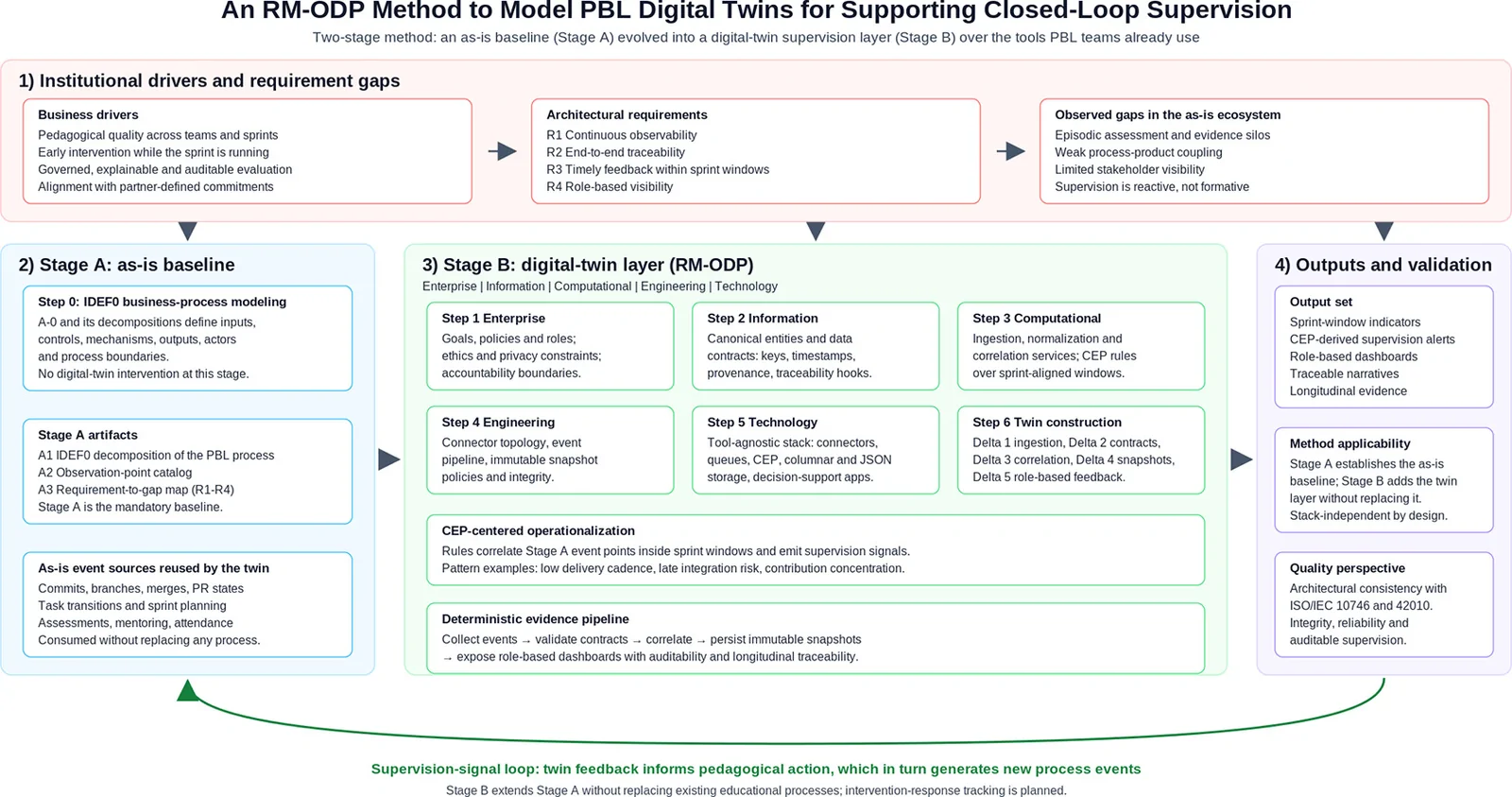

Specifications table:

**Table:** 

**Subject area**	Computer Science
**More specific subject area**	*Software Engineering Education; Learning Analytics; Digital Twin Architecture*
**Name of your method**	*RM-ODP-based PBL Digital Twin Modeling Method*
**Name and reference of original method**	*RM-ODP (ISO/IEC 10,746) and ISO/IEC/IEEE 42,010 architecture description applied to PBL observability*
**Resource availability**	*Modeling artifacts and example mappings (case instantiation)* Concrete artifact examples are reproduced in the article (Table 2, Table 3, Listings 1–2); measured indicators of the case instantiation are reported in Table 4, Table 5.

## Background

Project-Based Learning (PBL) emphasizes authentic and collaborative problem solving, yet its evaluation often relies on fragmented evidence collected through disconnected tools and episodic assessments [[1], [2], [3]]. In software engineering courses, traces of student activity are distributed across repositories, task boards, classroom interactions, and formal deliverables. This fragmentation makes it difficult to observe the learning process as it unfolds and to intervene in time when problems emerge [4]. When decisions depend on incomplete or delayed evidence, supervision tends to become reactive rather than formative.

PBL environments frequently attempt to emulate professional development contexts similar to industry internships. However, academic supervision in internships is typically episodic and dependent on declarative reports rather than operational evidence. In contrast, a PBL ecosystem supported by a Digital Twin can provide continuous observability based on events generated by the digital tools used during project execution. This approach enables supervision grounded in operational data while preserving the pedagogical structure of PBL programs.

The method presented in this article is motivated by operational supervision needs within PBL programs. Coordinators and instructors must monitor multiple teams working across several sprints, identify risks before sprint closure, justify evaluation outcomes under institutional governance, and maintain alignment with partner-defined project commitments. Supporting these decisions requires architectural capabilities that ensure continuous evidence capture, explicit causal links between process activities and outcomes, timely feedback delivery, and differentiated information visibility across stakeholders.

These needs can be expressed through four architectural requirements. R1 – Continuous observability, requiring persistent visibility into student engagement, collaboration patterns, and sprint progression. R2 – End-to-end traceability, ensuring that process events, produced artifacts, and evaluation decisions remain causally linked. R3 – Timely feedback delivery, enabling supervision signals to be generated during active sprint windows so that interventions can influence outcomes. R4 – Role-based visibility, providing differentiated views and access levels for students, instructors, coordinators, and external partners.

Current PBL ecosystems rarely satisfy these requirements consistently. Evaluations typically rely on episodic deliverables such as sprint reviews and final presentations, leaving large portions of the learning process unobserved [5]. Evidence is fragmented across multiple platforms, preventing integrated views of collaboration dynamics or project progression [6,7]. As a result, feedback frequently occurs after sprint completion, when corrective actions have limited pedagogical impact [4]. In addition, evaluation often focuses on final artifacts while neglecting the processes that produced them, such as task transitions, repository activity, and collaborative work patterns [2].

Digital twin architectures provide a potential response to these limitations. Digital twins are commonly defined as synchronized virtual representations of systems that enable monitoring, analysis, and optimization of their real-world counterparts [1]. The concept has been widely explored in manufacturing and industrial environments [8] and surveyed across multiple domains including cyber-physical systems and data-driven services [9,10]. By consolidating heterogeneous events and preserving traceability between activities and outcomes, a digital twin can support continuous monitoring and evidence-based supervision.

Beyond industrial settings, digital twins have also been explored in education, including web-based digital-twin environments for online engineering education [11], learning experiences built around digital twins of production systems [12], and course concepts probing the benefits and barriers of digital-twin technology in engineering curricula [13]. In parallel, the learning analytics community has consolidated reference models and architectures for collecting, processing, and acting on learner data [[14], [15], [16]], as well as event-centric interoperability specifications such as the Experience API (xAPI) for activity-stream collection [17]. These initiatives, however, typically focus on course-level analytics over learning-management-system data or on domain-specific simulation assets; they do not provide an architectural method for modeling the whole PBL ecosystem as a synchronized digital twin that supports closed-loop supervision. The method presented here addresses this gap by combining RM-ODP viewpoints with event-driven observability and CEP over the operational tools that PBL teams already use.

Applying digital twins to educational contexts, however, requires architectural rigor to ensure that heterogeneous events are captured and interpreted consistently. This work addresses that challenge by structuring a PBL Digital Twin using the five RM-ODP viewpoints—enterprise, information, computational, engineering, and technology—aligned with ISO/IEC 10,746 and ISO/IEC/IEEE 42,010 [18,19]. The method builds upon prior architectural work that positioned a Digital Twin for PBL supervision [20], extending it by formalizing a reproducible modeling procedure.

Within this architecture, Complex Event Processing (CEP) enables the correlation of events such as commits, task transitions, assessment submissions, and classroom interactions within sprint-aligned temporal windows. CEP mechanisms allow distributed operational events to be interpreted as supervision signals, supporting monitoring of collaboration patterns, cadence deviations, and artifact evolution within active sprint windows [[21], [22], [23]]. The method described in this article integrates these elements into a structured procedure for modeling PBL digital twins capable of supporting continuous and evidence-based supervision.

## Method details

The method is organized into two sequential and cumulative stages. Stage A establishes the baseline by identifying what already exists in the PBL ecosystem and where evidence is generated. Stage B represents an architectural evolution of that baseline, introducing Digital Twin capabilities to capture, correlate, and operationalize the same evidence through Complex Event Processing (CEP), while maintaining humans in the decision loop.

In the ongoing thesis cycle reported in this article, methodological emphasis is intentionally placed on the completeness of Stage A. Business-process decomposition, the event-point catalog, and requirement-to-gap mapping define the architectural invariants and observation boundaries that condition subsequent Digital Twin decisions.

**Stage A. As-is modeling:** The current PBL ecosystem is modeled using IDEF0 [24]. This step captures existing business processes, stakeholders, information flows, and supporting tools such as learning management systems, version-control platforms, and task boards exactly as they operate today, without introducing any Digital Twin capability. The as-is model makes explicit which events are already produced by the ecosystem, including commits, task transitions, assessment submissions, and attendance records, as well as the systems in which these events reside.

**Requirements-driven gap analysis:** The RM-ODP viewpoints [19] are applied to the as-is model in order to evaluate whether the architectural requirements R1–R4 are satisfied. This analysis identifies observation points that remain invisible or fragmented. These include events that are produced but not captured, stakeholder concerns that lack role-appropriate evidence, and feedback loops that remain open.

**Fig. 1:**
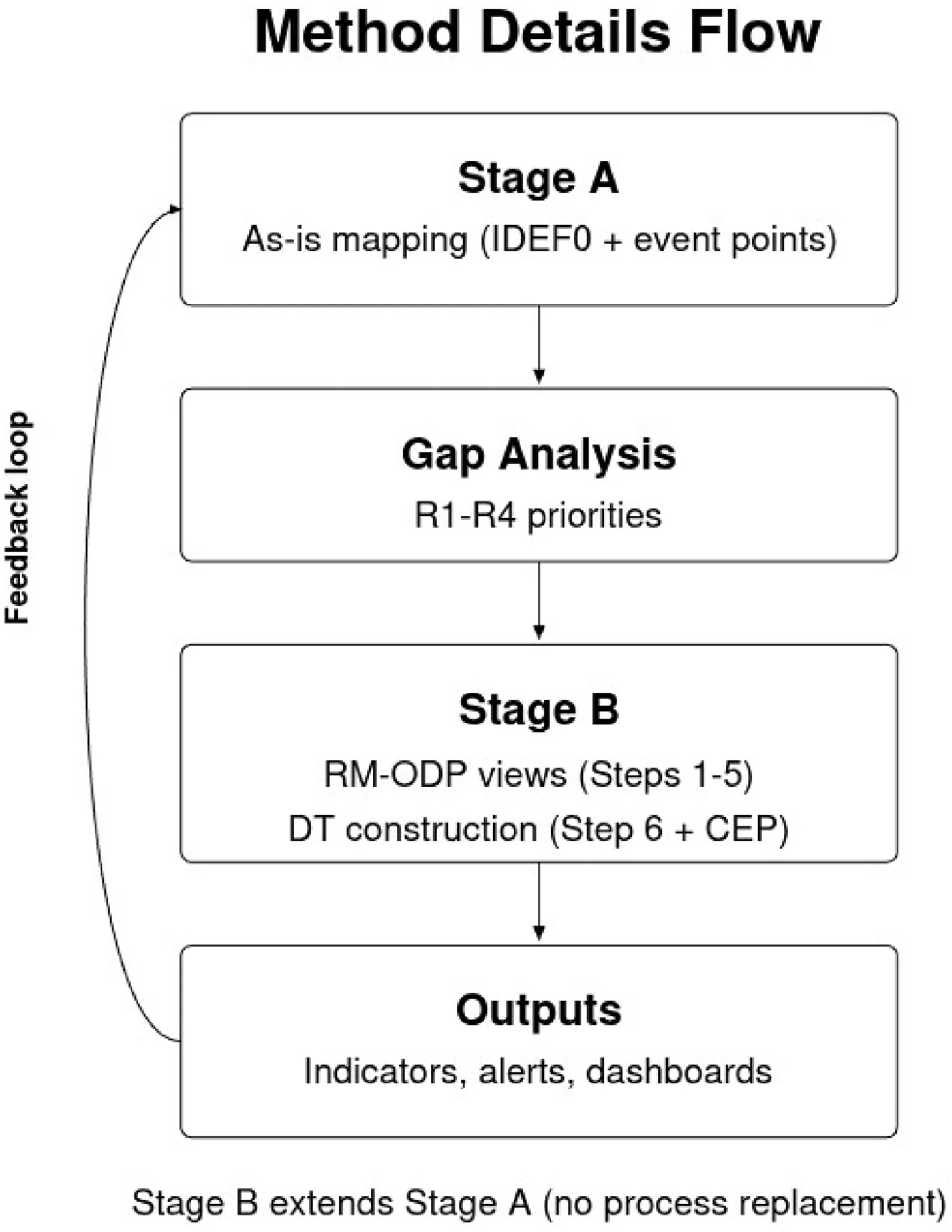
Method flow illustrating the relationship between Stage A and Stage B. Stage A establishes the as-is.

This requirement-to-gap mapping connects Stage A to Stage B by defining what the Digital Twin must observe first and what remains outside the current implementation scope. In Fig. 1, this corresponds to the gap analysis block located between Stage A and Stage B.

**Stage B. Digital Twin modeling:** Stage B starts from the artifacts produced in Stage A, namely the as-is model and the requirements-driven gap map. These artifacts are evolved into a to-be architecture. The method preserves the entire as-is ecosystem and introduces the Digital Twin as an additional architectural layer. This layer operates as a cybernetic component that reads events already produced by the as-is processes, correlates them through CEP, and delivers feedback to stakeholders within the active sprint window.

The Digital Twin does not replace or modify existing systems. Instead, it observes and augments them. Each RM-ODP viewpoint is applied to specify how the Digital Twin captures events in the enterprise viewpoint, normalizes them in the information viewpoint, correlates them in the computational viewpoint, distributes them in the engineering viewpoint, and implements them in the technology viewpoint. A final consolidation step defines the Digital Twin itself, including its cybernetic supervision capabilities, incremental evolution, and operational outputs.

In the current cycle, Stage B is instantiated with uneven maturity across viewpoints. Enterprise and information artifacts are already stabilized for repository evidence. Computational, engineering, and technology artifacts are operational for the implemented repository-ingestion path and remain open for expansion to additional PBL evidence channels.

Each step produces modeling artifacts that together specify a PBL Digital Twin ready for implementation. The key architectural principle is evolutionary continuity from Stage A to Stage B. The as-is layer remains the source of events, while the Digital Twin acts as the consumer and correlation layer added on top of it. In this way, Stage B extends Stage A without altering the original processes.

**Business process modeling with IDEF0:** The PBL business process is first modeled using IDEF0 in order to define inputs, controls, mechanisms, and outputs according to the IDEF0 syntax and semantics standard [24]. The A-0 systemic diagram and the decomposed diagrams capture the institutional context, including curriculum guidelines, TAPI, and partner constraints, together with the operational flow of sprints, instruction, and review cycles.

This step clarifies the process boundaries and creates the baseline from which critical observation points are derived. In Fig. 1, this step corresponds to the Stage A block.

**Enterprise viewpoint (goals, policies, roles):** The enterprise viewpoint uses the IDEF0 model to define pedagogical goals, institutional policies that constrain observation, and the responsibilities of each stakeholder.

In practice, this step clarifies learning outcomes and supervision goals, formalizes ethics and privacy constraints, and establishes accountability boundaries among students, mentors, coordinators, and external partners. This definition determines the observation scope and justifies which events will be captured later. In Fig. 1, this step is located inside Stage B.

**Information viewpoint (concepts, data contracts):** The information viewpoint specifies the conceptual model and the data contracts used to normalize heterogeneous PBL evidence. Each event contract carries identity keys such as course, cohort, group, project, and artifact. It also includes temporal markers such as event time, ingestion time, and window scope, operational semantics including event type, source system, and phase linkage, and traceability hooks identifying actors and artifacts. These contracts define the canonical vocabulary, preserve traceability across institutional contexts, and enable interoperability among platforms. In Fig. 1, this step is located inside Stage B.

**Computational viewpoint (services, events, CEP):** The computational viewpoint defines the logical services and event-processing rules. Services are separated into ingestion, normalization, correlation, and analytics in order to maintain modular and auditable event handling. CEP rules are bound to the critical event points identified in Stage A. Windowing strategies aligned with PBL milestones correlate patterns that signal risks or learning opportunities. These correlations generate feedback triggers such as alerts, dashboards, or summaries. In this way, the signal side of the closed supervision loop is operationalized by linking end-to-end process evidence to timely pedagogical actions within the active sprint window [[21], [22], [23]]; closing the loop on the intervention side remains a human responsibility that the method supports but does not automate.

In Fig. 1, this step is located inside Stage B. In the Inteli instantiation, a first executable subset of rules was defined for repository evidence and sprint cadence supervision, and scenario examples are reported in the Method application section in Table 1.

**Table 1:** Instantiated CEP rule scenarios in the Inteli repository-evidence cycle.

Scenario	Correlated event points (Stage A origin)	Temporal window	Derived output for supervision
Low delivery cadence	Sprint-planning backlog creation + commit stream + task transition updates	Rolling 3–5 days inside the active sprint	Alert indicating insufficient progress rhythm, with trace links to commits and backlog items for professor review
Late integration risk	Planned delivery date (TAPI or sprint plan) + branch and merge events + pull request closure state	Milestone-centered window (T-2 to T + 1 days)	Risk signal indicating probable delivery slippage, including unresolved integration points and missing merge evidence
Contribution concentration	Team roster + commit authorship + artifact-touch distribution across repository paths	Whole sprint window with daily refresh	Indicator of high concentration or low collaboration balance, supporting mentoring action on team work distribution

**Engineering viewpoint (distribution and pipeline):** The engineering viewpoint defines how services are distributed and integrated so that temporal integrity is preserved. Connectors are implemented as adapters for version-control systems, learning management systems, and task boards.

The pipeline is structured in layers composed of ingestion, validation, enrichment, and storage. Snapshot isolation is enforced so that events remain immutable and auditable. Engineering also defines algorithm-driven control meshes aligned with PBL evaluation requirements. These controls associate planned delivery schedules with study events, submissions, planned dates, and actual delivery dates. Logical consistency rules evaluate team activity sufficiency based on workload, cadence, and milestone progression. The resulting signals feed supervision flows that support timely human intervention. In Fig. 1, this step is located inside Stage B.

**Evidence persistence and integrity:** To preserve temporal integrity and prevent the loss of historical evidence, events are stored as immutable snapshots. Each snapshot contains structured data such as version-control events and derived indicators stored in columnar formats together with metadata including snapshot identifiers, time intervals, and provenance.

Snapshot isolation provides auditability and supports longitudinal analyses even when source systems are rewritten or altered. The method is storage-agnostic.

**Technology viewpoint (platform choices):** The technology viewpoint specifies implementation choices and reference stacks without constraining the method. Typical components include connectors for event capture in version-control platforms, learning management systems, and task systems; object storage with columnar or JSON formats and versioned metadata for persistence; and a decision-support application for analytics and reporting.

The method remains tool-agnostic so institutions can adopt technologies that fit their governance and infrastructure constraints. In Fig. 1, this step is located inside Stage B. This step maintains the portability of the method while documenting the minimum platform capabilities required for implementation.

**Digital Twin construction:** This step consolidates the outputs of the previous viewpoints into the Digital Twin itself. The Digital Twin becomes a cybernetic layer for the PBL ecosystem and provides evidence-based support to all stakeholders, from students to faculty. The construction is organized into incremental deltas, each adding a well-defined capability. In Fig. 1, this step corresponds to the final part of Stage B and leads to the system outputs.

• **Delta 1.** Event ingestion baseline. Connectors defined in the engineering viewpoint are assembled into an operational pipeline that captures events from all source systems identified in the as-is model.
• **Delta 2.** Contract enforcement. Data contracts defined in the information viewpoint are activated as validation rules. These rules ensure that every ingested event conforms to the canonical vocabulary before entering the Digital Twin.
• **Delta 3.** CEP correlation. The CEP rules defined in the computational viewpoint are deployed over the validated event stream. This processing produces derived indicators, pattern detections, and alerts aligned with sprint time windows [[21], [22], [23]].
• **Delta 4.** Evidence persistence. Immutable snapshots are generated and stored according to the engineering policies. This guarantees auditability and enables longitudinal analysis across projects and cohorts.
• **Delta 5.** Cybernetic feedback. Analytics and visualization services are activated as an integral Digital Twin capability. These services generate indicators aligned with the RM-ODP viewpoints [19], including policy compliance indicators in the enterprise viewpoint, data completeness indicators in the information viewpoint, and event-derived operational signals in the computational viewpoint.

The Digital Twin delivers customized dashboards, alerts, and explanatory narratives tailored to each stakeholder role. A dedicated decision-support interface or equivalent software supports supervision within active sprint windows and comparisons across teams, sprints, and courses. Access and visibility levels follow the policies defined in the enterprise viewpoint. For example, students see indicators related to their own team progress, while coordinators can access cross-team metrics and aggregated program-level indicators. Final decisions remain under human responsibility.

After this step, the Digital Twin produces several outputs. These include sprint-window indicators of student engagement, collaboration cadence, and artifact quality; alerts triggered by CEP rules when patterns deviate from expected sprint behavior; longitudinal evidence traces that support formative and summative evaluation; and dashboards tailored to each stakeholder role, including supervising professors, coordinators, partners, and students. Detection and prediction are cybernetic processes automated by the Digital Twin, while pedagogical and administrative decisions remain human, preserving accountability and professional judgment.

**Fig. 2:**
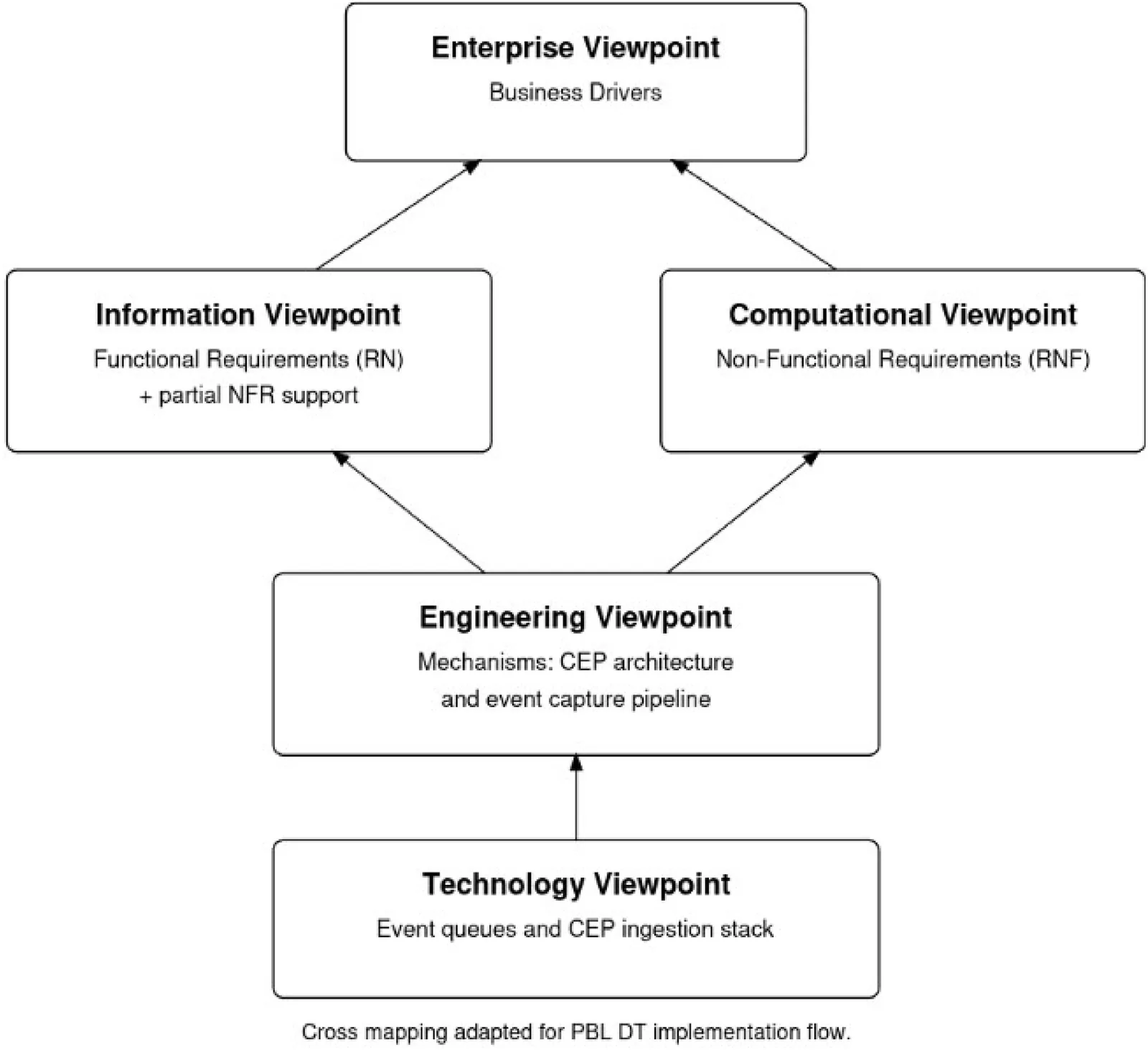
Cross-shaped mapping of RM-ODP viewpoints for implementation aspects. The enterprise viewpoint is translated into business drivers. The information viewpoint emphasizes functional requirements (RN) while also covering part of the non-functional concerns. The computational viewpoint emphasizes non-functional requirements (RNF). These requirement views are integrated in engineering mechanisms, including the CEP architecture and event capture infrastructure, and are then realized in the technology viewpoint through concrete components such as event queues and CEP ingestion services. This progression shows how the Digital Twin is grounded from business intent to executable infrastructure, following the architecture-oriented interpretation discussed in [5].

**Implementation aspects:** Architecture definition and artifact production are incremental activities that begin in the first step of the method rather than appearing only at the end. The initial step establishes the as-is architectural baseline, including process boundaries, actors, systems, and event points. The subsequent steps progressively refine and implement the to-be Digital Twin layer. In this sense, implementation aspects are continuously specified and consolidated throughout the method while preserving traceability between business-process modeling and technical realization. Fig. 2 explains the Digital Twin foundation path adopted in this method. First, the enterprise viewpoint defines the business drivers that justify supervision goals and architectural priorities. Second, these drivers are translated into requirement views. The information viewpoint structures functional requirements and data semantics, while the computational viewpoint structures non-functional behavior such as latency, integrity, and reliability, with partial overlap through the information contracts. Third, both requirement views are materialized in engineering mechanisms centered on Complex Event Processing and end-to-end event capture [[21], [22], [23]]. Finally, the technology viewpoint implements these mechanisms through concrete components including event queues, connectors, and CEP ingestion and processing services. Through this sequence, the Digital Twin is not introduced as an isolated artifact but is progressively constructed from business rationale to operational event-processing infrastructure, following the RM-ODP architectural organization and architecture-description principles adopted in this work [18,19].

The resulting architectural view maps the method steps into coherent functional blocks:

• Event capture. Connectors and adapters collect events from the as-is source systems such as version-control platforms, learning management systems, and task boards. These mechanisms follow the enterprise policies and engineering distribution defined earlier in the method.
• Normalization (Step 2). Contract validation services enforce the data contracts and canonical vocabulary defined in the information viewpoint.
• Correlation and CEP (Step 3). Event processing services apply the windowing strategies, pattern matching, and aggregation rules defined in the computational viewpoint in order to derive indicators and alerts.
• Persistence (Step 4). Immutable snapshot storage preserves temporal integrity and auditability as specified in the engineering viewpoint.
• Cybernetic feedback (Step 6). Analytics and visualization services deliver sprint-window dashboards, alerts, and explanatory narratives to stakeholders, as consolidated in the Digital Twin construction step.

The core data model follows a canonical hierarchy that preserves institutional traceability: course, module, sprint, group, student, and artifacts such as backlog items, repository objects, and assessment records. Relationships bind each event to its institutional context, enabling aggregation by sprint, team, or cohort while maintaining auditability.

The evidence pipeline follows a deterministic sequence aligned with the deltas defined in the Digital Twin construction step. First, source events are collected from the as-is systems. Second, events are validated against data contracts and enriched with canonical keys and time markers. Third, CEP rules correlate events within sprint windows to produce indicators and alerts. Fourth, both raw events and derived indicators are stored as immutable snapshots. Finally, cybernetic feedback is delivered to stakeholders through role-specific dashboards and alerts, while all decisions remain human.

The method described in this work was structured to allow replication in other PBL environments. Replication is enabled by the separation between the observational baseline (Stage A) and the Digital Twin architectural evolution (Stage B), as well as by the definition of artifacts produced at each step of the procedure. The following sequence summarizes the steps required to reproduce the method:

1. Model the PBL ecosystem. Identify the institutional context and represent the PBL operational processes using IDEF0, capturing actors, inputs, controls, mechanisms, and outputs. This produces the as-is process model that defines the boundaries of the ecosystem.
2. Identify operational event points. Analyze the as-is process model to determine where operational evidence is generated across systems such as version-control platforms, learning management systems, task boards, assessment tools, and classroom activities. This step produces the event-point catalog.
3. Evaluate architectural requirements. Apply the requirements R1–R4 to the as-is model and perform requirement-to-gap mapping in order to identify which supervision needs are satisfied and which observation points remain invisible or fragmented.
4. Define the enterprise viewpoint. Establish the governance model of the Digital Twin, including learning goals, institutional policies, privacy constraints, and the roles and responsibilities of stakeholders such as students, instructors, coordinators, and partners.
5. Specify the information viewpoint. Design the conceptual model and data contracts that normalize heterogeneous PBL events. These contracts define canonical identifiers, temporal attributes, event types, and traceability relationships between actors, artifacts, and activities.
6. Define the computational viewpoint. Specify the event-processing services and the Complex Event Processing (CEP) rules used to correlate events within sprint-aligned temporal windows and generate supervision signals.
7. Design the engineering viewpoint. Define the distributed event pipeline, including connectors, validation mechanisms, enrichment steps, and immutable event persistence policies.
8. Define the technology viewpoint. Select or document the technological components required to implement the event ingestion, event processing, and storage infrastructure, while keeping the method independent from specific platforms.
9. Construct the Digital Twin layer. Integrate the artifacts produced in the previous steps into an operational Digital Twin capable of ingesting events, enforcing data contracts, correlating patterns through CEP, and delivering role-specific feedback to stakeholders.

Following these steps allows institutions to reproduce the method independently of their technological stack. Because the procedure relies on architectural viewpoints and event-driven observation rather than on specific tools, different implementations can adopt alternative platforms while preserving the same methodological structure. The resulting artifacts—including the process model, event catalog, requirement–gap map, RM-ODP architectural views, and CEP rules—constitute a reproducible specification for constructing a PBL Digital Twin capable of supporting continuous supervision and evidence-based evaluation.

### Method application: Inteli PBL case

We apply the method to the Inteli PBL curriculum, a higher-education program centered on Project-Based Learning in engineering and computing, with industry partners and assessment focused on process as well as final artifacts. The curriculum is interdisciplinary and tool-intensive, which increases the need for observability. Mentoring and intervention depend on evidence scattered across repositories, task boards, classroom interactions, and formal assessments. A digital twin provides a structured way to connect institutional goals, learning evidence, and operational events.

The Inteli PBL process is organized into a 10-week module with five two-week sprints. Each module is formalized by a TAPI (Institutional Project Charter, from Portuguese Termo de Abertura do Projeto Institucional), defining the problem, learning outcomes, scope, evaluation criteria, and ethical constraints. Teams follow Scrum-inspired routines including planning sessions, reviews, and continuous mentoring. Roles include Product Owner (PO) and Scrum Master (SM), supported by a supervising professor and domain instructors. External partners provide authentic problems and participate in feedback cycles.

**Fig. 3:**
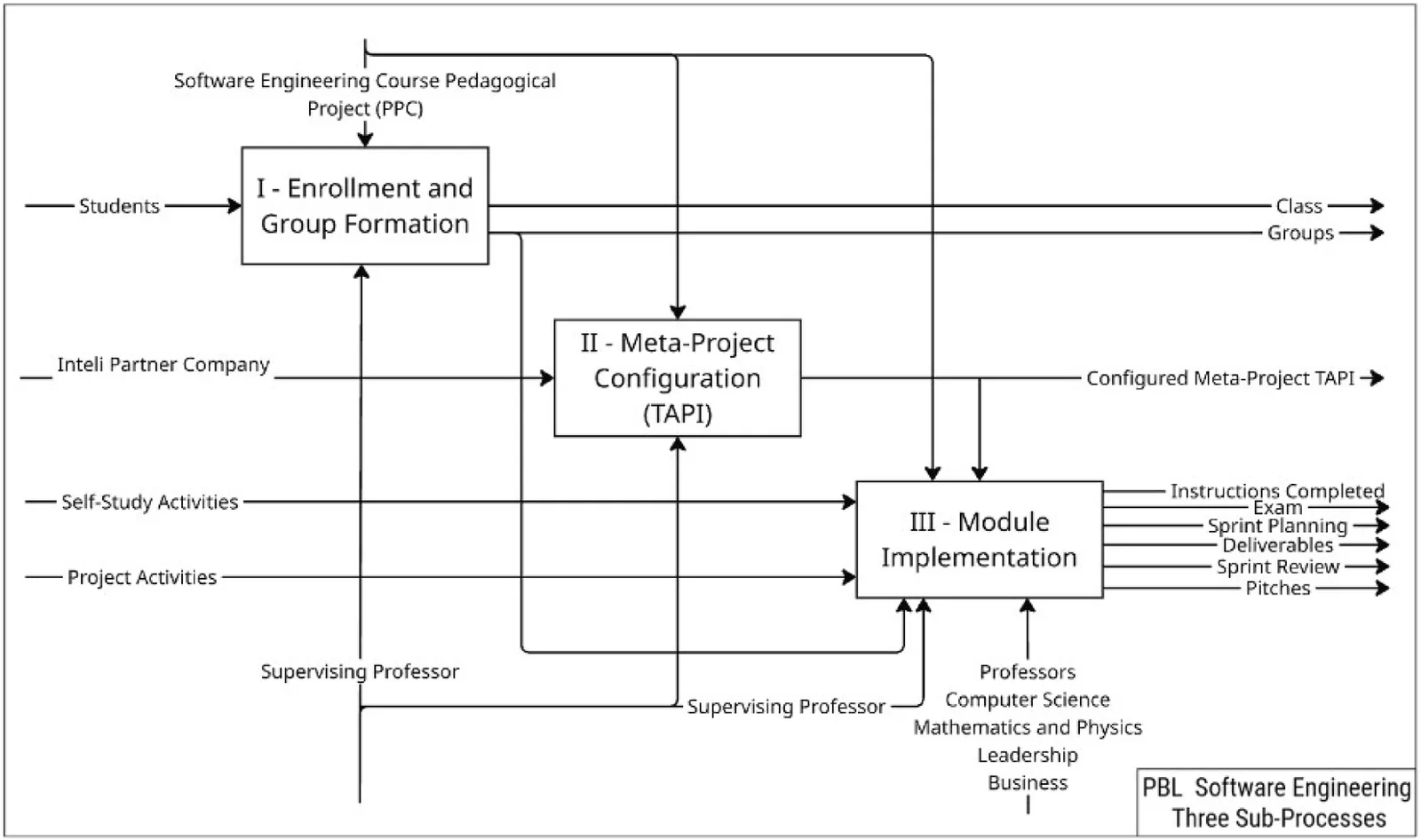
IDEF0 as-is model of the Inteli PBL ecosystem showing three sub-processes, principal actors, and key variables. Adapted from our ACDSA 2026 conference paper (IEEE publication track) [20].

The business process is first modeled in IDEF0 using ICOM (Inputs, Controls, Mechanisms, Outputs) to make process boundaries explicit. The as-is IDEF0 decomposition of the Inteli PBL ecosystem, shown in Fig. 3 separates three main sub-processes, originally presented in the ACDSA 2026 conference paper by Arakaki, da Silva, and Brandão.

Sub-process I (Enrollment and Group Formation) receives students and produces classes and project groups under the control of the Software Engineering Course Pedagogical Project (PPC).
Sub-process II (Meta-Project Configuration) integrates the partner company and the supervising professor in order to produce the configured TAPI.
Sub-process III (Module Implementation) is the most event-intensive stage. It consumes self-study activities, project activities, and instructor inputs, and produces completed instructional activities, exams, sprint planning records, deliverables, sprint reviews, and project pitches.

The principal actors students, supervising professors, partner companies, and domain instructors from Computer Science, Mathematics and Physics, Leadership, and Business appear as inputs and mechanisms in the IDEF0 model. This as-is model constitutes the event source that the Digital Twin observes.

Inputs include student actions and project artifacts. Controls include institutional guidelines, TAPI constraints, and assessment policies. Mechanisms include learning management systems, version-control platforms, task boards, and teaching staff. Outputs include graded deliverables, feedback records, and evidence traces. These IDEF0 models define where evidence is produced and where observation is required.

In methodological terms, the present article anchors its strongest empirical grounding in this Stage A layer. The IDEF0 decomposition and observation-point mapping are complete and serve as the reference baseline for every Stage B implementation decision in the current cycle.

RM-ODP viewpoints are then used to identify critical observation points. These points are encoded as event types and data contracts. CEP rules attach to these events and transform distributed evidence into indicators that reveal process status, risks, or deviations from the expected sprint cadence.

The digital twin enforces a clear separation between cybernetic and human responsibilities. Detection of patterns and prediction of risks are automated by CEP rules operating over the event stream. Pedagogical and administrative decisions remain with human stakeholders including supervising professors, coordinators, and industry partners, who act on dashboards, alerts, and explanatory narratives derived from the twin. This separation closes the supervision loop while preserving professional judgment, accountability, and institutional governance.

An illustrative scenario occurs during sprint planning. Backlog items are created and linked to the TAPI. CEP then correlates planning events with subsequent repository activity and task transitions. If a team shows low task movement and sparse code contributions within the expected sprint window, the system raises an alert supported by evidence traces. The supervising professor reviews the evidence, decides whether intervention is necessary, and records the action. This intervention becomes a new event in the system and closes the feedback loop.

To illustrate this operational cycle without shifting the focus away from the method, a dynamic scenario called Feature Delivery Loop is used as a supporting example for the CEP artifact. The primary artifact remains the CEP correlation specification defined in the computational viewpoint and operationalized through the instantiated rule set summarized in Table 1.

**Fig. 4:**
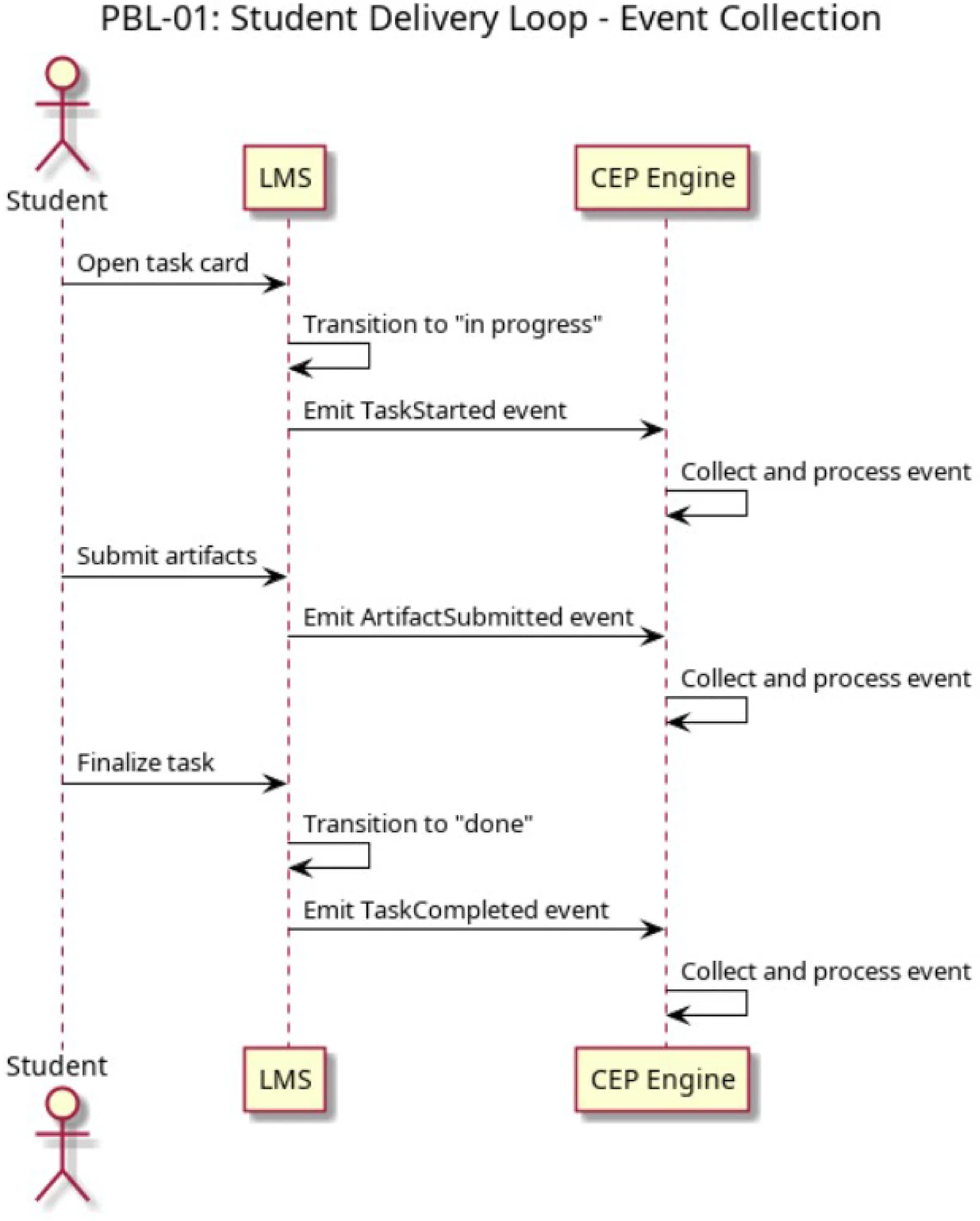
Dynamic scenario SE-01 (Feature Delivery Loop) used as support for the CEP artifact in the Inteli case. Native PBL events including planning actions, repository activity, and task-flow transitions are collected and correlated by CEP within sprint windows, illustrating how rule execution generates supervision signals within the active sprint window for human intervention. Adapted from the ACDSA 2026 conference publication (IEEE publication track) [20].

In this scenario, illustrated in Fig. 4, native PBL events including planning actions, repository activity, and task flow transitions are collected and correlated by CEP within sprint windows. Rule execution produces supervision signals within the active sprint window that support human intervention.

To maintain CEP as a tangible artifact rather than a purely narrative description, the instantiated rule scenarios currently used in the repository-evidence Digital Twin path are summarized in Table 1. These scenarios link observation points, temporal windows, and supervision outputs.

An operational repository snapshot algorithm is also implemented in the Inteli case. This algorithm runs in the repository-ingestion pipeline in order to prevent evidence loss and preserve longitudinal traceability. During each collection cycle the algorithm performs the following actions:

1. Identifies the target repository and the reference time interval corresponding to the sprint or module window.
2. Captures a full repository state descriptor including the HEAD commit, branch set, tags, commit graph segment, and file-tree hashes.
3. Extracts incremental events since the previous snapshot, including commits, authorship information, timestamps, file diffs, and merge metadata.
4. Binds all records to canonical keys such as course, module, sprint, team, and artifact.
5. Persists an immutable snapshot package containing a unique snapshot identifier, capture timestamp, source provenance, and integrity hash.

Because each snapshot package is append-only and time-stamped, later repository rewrites, branch deletions, or force-push operations do not erase prior evidence already captured by the Digital Twin.

As part of the Inteli case, the method was instantiated as a repository-evidence Digital Twin experiment that not only modeled the architecture but also specified, implemented, and integrated the event-processing path used for supervision.

**Stage A artifacts (baseline):**

A1. IDEF0 as-is decomposition artifact. A-0 and decomposed process views for enrollment and group formation, meta-project configuration, and module implementation.
A2. Observation-point catalog. Explicit list of native event sources in the operational process including version-control actions, task transitions, assessments, attendance, and mentoring interactions. An excerpt is reproduced in Table 2.

**Table 2:** Observation-point catalog excerpt (artifact A2) as instantiated at Inteli.

Observation point	Source system (as-is)	Canonical event types	Primary contract keys
Code contribution	Version-control platform	repo.commit.pushed; repo.branch.merged; repo.mr.opened/closed	course, module, sprint, group, author (pseudonym), artifact path
Task flow	Task board	task.card.created; task.card.transitioned	course, module, sprint, group, card id, state transition
Instructional activity	Learning management system	activity.snapshot; selfstudy.completed; ceremony.attended	course, module, sprint, group, student (pseudonym), activity id
Formal delivery	Assessment / project charter (TAPI) workflow	deliverable.submitted; deliverable.evaluated	course, module, sprint, group, deliverable id (D1–26…D5–21), competency axis
A3. Requirement-to-gap map. Linkage between observed process limitations and requirements R1 to R4, establishing priorities for Digital Twin implementation deltas. An excerpt is reproduced in Table 3.

**Table 3:** Requirement-to-gap map (artifact A3) for the Inteli case.

Requirement	Gap observed in the as-is ecosystem	Stage B artifacts	Status in current cycle
R1 – Continuous observability	Sprint progress visible only at reviews; repository activity not continuously observed	B3, B4, B6	Implemented for repository evidence
R2 – End-to-end traceability	No causal link between process events, artifacts, and evaluation decisions; evidence lost on repository rewrites	B2, B4	Implemented (immutable snapshots)
R3 – Timely feedback	Feedback delivered only after sprint closure	B3, B6	Implemented for repository evidence (batch correlation)
R4 – Role-based visibility	Undifferentiated views; no role-scoped evidence	B1, B6	Partially implemented

**Stage B artifacts (implementation):**

B1. Enterprise artifact. Stakeholder and role matrix including students, supervising professors, partners, and coordination. This artifact defines supervision goals and policy constraints derived from the TAPI and institutional governance.
B2. Information artifact. Canonical entity model including course, module, sprint, group, student, TAPI, backlog, and artifact. Event contracts include identity keys, timestamps, provenance, and traceability attributes. A concrete contract instance is given in Listing 1.
B3. Computational artifact. Service decomposition including ingestion, normalization, CEP correlation, and feedback services. A sprint-aligned CEP rule set supports cadence monitoring and risk detection. Executable rule specifications are given in Listing 2.
B4. Engineering artifact. Connector topology and event pipeline composed of ingestion, validation, enrichment, and storage layers, together with an immutable snapshot policy that preserves temporal integrity.
B5. Technology artifact. Executable stack including connectors, queue-based event flow, CEP execution components, columnar and JSON persistence layers, and decision-support interfaces.
B6. Digital Twin integration artifact. Repository-ingestion experiment producing sprint-window indicators of code cadence, task movement, and alert triggers used to support supervisory intervention.

#### Concrete artifact examples

To improve reproducibility, this subsection reproduces concrete excerpts of four artifacts that adopters of the method must produce: the observation-point catalog (artifact A2, Table 2), the requirement-to-gap map (artifact A3, Table 3), an event data-contract instance (artifact B2, Listing 1), and executable CEP rule specifications (artifact B3, Listing 2). Values are those of the Inteli instantiation; identifiers are pseudonymized and the contract instance is illustrative of the enforced schema.

Listing 1. Event data-contract instance enforced in the repository-evidence cycle (illustrative values; pseudonymized identifiers).

{

"event_type": "repo.commit.pushed",

"source": "version-control",

"course": "software-engineering", "module": "M-2026–2A",

"sprint": 3, "group": "gA",

"author": "sha256:a3f9c21b04e7d8aa",

"artifact": "src/backend/routes/auth.js",

"event_time": "2026–05–14T21:37:02Z",

"ingestion_time": "2026–05–15T08:00:11Z",

"window_scope": "sprint-3",

"provenance": {"repo": "r1", "branch": "feat/auth", "commit": "9c4e…"},

"integrity_hash": "sha256:…"

}

Listing 2. Executable CEP rule specifications for two scenarios of Table 1. The cramming_risk threshold (>50% of commits in the final 20% of the window) is the value used in the first-generation Inteli deployment [25].

RULE low_delivery_cadence.

SCOPE (group, sprint).

WINDOW rolling(5 days) WITHIN sprint.

WHEN count(task.card.transitioned) < θ_tasks.

AND count(repo.commit.pushed) < θ_commits.

AND count(backlog.item.created SINCE sprint_planning) > 0.

EMIT alert(level = warning, target = supervising_professor,

trace = [commits, cards, backlog_items])identifiers).

RULE cramming_risk.

SCOPE (group, sprint).

WINDOW sprint.

WHEN share(repo.commit.pushed, last 20% of window) > 0.50.

EMIT risk(pattern = CRAMMING_RISK, target = monitor,identifiers).

trace = [commit timeline]).

**Gap-to-implementation consolidation:**

R1 (continuous observability) is addressed by artifacts B3, B4, and B6 through continuous repository ingestion, CEP processing windows, and live indicators.
R2 (traceability) is addressed by artifacts B2 and B4 through contract-enforced identifiers and immutable snapshots that link events, artifacts, and supervision decisions.
R3 (timely feedback) is addressed by artifacts B3 and B6 through CEP correlations and alert or dashboard outputs produced during active sprint windows.
R4 (role-based visibility) is partially addressed in this cycle by artifacts B1 and B6 through role-scoped outputs. Full institutional coverage remains an explicit next increment.

This consolidation demonstrates that the Inteli experiment operationalized the full Stage A and Stage B chain, producing explicit artifacts from business-process modeling to a running Digital Twin supervision service. In the current cycle this chain is operational for the repository-evidence path; the maturity of every capability is classified explicitly in Table 5.

### Method validation

Validation was conducted through the Inteli PBL case as an ongoing implementation cycle, not only as conceptual modeling. In the current cycle, a Digital Twin oriented system is being coded and integrated in order to capture data and events from Git repositories associated with PBL artifact deliveries in each sprint. Commit-level and repository-level evidence are transformed into supervision signals delivered within the sprint window. The validation combines qualitative evidence — supervision signals and their pedagogical use — with quantitative indicators measured on the institutional case, reported below.

Two distinct loops should be separated when reading these claims. The supervision-signal loop runs from operational events to contract-validated evidence, correlation over sprint windows, and role-scoped signals delivered to faculty while the module is still running; this loop is demonstrated in the current cycle. Within it, correlation is executed as batch replay over sprint windows rather than as a continuously running streaming engine, so that timeliness in this article means delivery inside the active sprint window, bounded by the ingestion cadence, and not sub-second latency. The intervention-response loop, which would record how a supervision signal changed pedagogical practice and feed that outcome back into the twin, is specified by the method but is not yet instrumented, and Table 5 classifies it as planned. For this reason the method is presented as an architecture for supporting closed-loop supervision, and every statement about closure in this article refers to the signal loop unless the text states otherwise.

The validation closes the requirement and gap chain presented earlier by explicitly mapping each identified gap to implemented or in-progress artifacts.

• Evidence-based evaluation gap (R1, R2). Addressed through continuous repository-event ingestion, contract-based identifiers, and immutable snapshots that link process evidence to delivered artifacts. The case already produces stable and comparable evidence traces across teams and sprints.
• Stakeholder performance visibility gap (R4). Addressed in progress through role-scoped outputs for students, supervising professors, and coordination roles, combined with policy constraints defined in the enterprise artifacts. This requirement is partially satisfied in the current cycle and remains under incremental expansion to full stakeholder coverage.
• Timeliness gap (R3). Addressed through CEP-oriented event correlation within sprint windows. Alerts and indicators are generated during active sprint execution rather than only after sprint closure. The case demonstrates the operational feasibility of timely feedback for pedagogical intervention.
• Process–product disconnect (R1, R2). Addressed by integrating process events such as contribution cadence and task-linked repository activity with artifact evolution. This integration allows evaluation to consider both what was delivered and how the delivery process occurred.

From a methodological perspective, the Inteli case confirms that the Stage A and Stage B chain is executable. Stage A provides the event baseline and the requirement-to-gap mapping, while Stage B implements the architectural mechanisms including data contracts, CEP correlation, immutable snapshots, and role-based feedback services that operationalize these gaps as concrete Digital Twin capabilities.

#### Quantitative and qualitative evidence from the Inteli instantiation

The Inteli case has been instantiated in three increments, and each increment was measured with evidence proportional to its maturity. The first-generation system is deployed institutionally at Inteli, where PBL spans all undergraduate programs and involves approximately 40 faculty members; its published validation used an LGPD-safe simulated dataset (37 projects, 459 project–member links, 103 commits), measured a deterministic processing time of 526.5 ms for five teams (105.3 ms per project, on a benchmark of 58 commits, 8 merge requests, 27 issues, and 20 document snapshots), operates on a daily ingestion cadence that bounds detection latency at 24 h, and executes a CEP set of nine team-level pattern categories in four families [25]. The second-generation event backbone was verified with a synthetic fixture: 3744 CloudEvents published, 3743 persisted, and 1 event rejected to a dead-letter queue by contract validation — the intended behavior of the Delta 2 contract-enforcement mechanism — together with 2884 activity snapshots. The third-generation repository-evidence cycle processed real, pseudonymized data of one undergraduate team end-to-end; its measured indicators are consolidated in Table 4.

**Table 4:** Measured indicators of the repository-evidence cycle (real, pseudonymized data of one institutional team).

Indicator	Measured value	Data regime
Corpus	3 team repositories; 11 pseudonymized contributors; 658 commits collected, 410 within the 10-week module window (5 sprints)	Real, pseudonymized
Privacy gate	0 cleartext personal identifiers after pseudonymization (automated scan; truncated SHA-256 identities)	Real
Derived delivery evidence	5 sprint delivery deltas; 23 persisted semantic delivery analyses; 249 records across 19 columnar (Parquet) tables	Real
Charter (TAPI) adherence index	100, 100, 98, 95, 93% across sprints 1–5, computed over 18 charter deliverables	Real
Requirements confrontation	84 declared requirements confronted with 10 board-expected requirements; 13 sprint-level confrontation records	Real
Supervision signals produced	10 evidence-anchored questions addressed to the faculty board	Real
Semantic-analysis latency	≈82–478 s per sprint analysis, depending on the local model (4–14B parameters; single 12 GB consumer GPU; no cloud services)	Measured
Analyzer promotion gate	Golden-set falsifiability gate requiring precision ≥ 0.90 and recall ≥ 0.85 against persona-based reference analyses; the promoted configuration passed all checks	Measured

Qualitatively, the supervision signals of the current cycle take the form of evidence-anchored questions to the faculty board rather than grades. For example, in Sprint 3 the twin asked how write routes would be protected by authentication, surfacing a security expectation introduced by a charter amendment valid precisely from that sprint onward; in Sprint 5 it flagged that the pitch deliverable had no planning or execution evidence. In the first-generation deployment, outputs are role-differentiated: monitors receive early alerts, specialist professors receive per-deliverable assessments, and advisors receive separate reports for individual assessment [25]. Systematic records linking these signals to specific pedagogical interventions are not yet collected: an evaluator-feedback study is in progress, and closed-loop intervention tracking is classified as planned work in Table 5.

**Table 5:** Implementation status of method capabilities in the current cycle.

Capability	Status	Evidence / scope
Stage A artifacts (IDEF0 as-is model, observation-point catalog, requirement-to-gap map)	Implemented	Complete for the Inteli case (Figs. 1, 3; Table 2, Table 3)
Repository event ingestion with immutable snapshots	Implemented	Real-data cycle (Table 4)
Data contracts and privacy gates	Implemented	Contract-based rejection (event backbone); zero-PII gate (real-data cycle)
Semantic delivery analysis with local models	Implemented (offline)	23 persisted analyses; promotion gate (Table 4)
CEP rule execution	Partially implemented	Executable engine exercised in the first-generation deployment (simulated data) [25]; in the current cycle, correlation runs as batch replay over sprint windows, not as a streaming engine
Multi-source integration (LMS, task boards, assessments)	Partially implemented	Event backbone verified with synthetic fixtures only; the repository channel is the operational path
Role-based visibility (full stakeholder coverage)	Partially implemented	Team-level and faculty-board views operational; full institutional coverage is the next increment
Closed-loop intervention tracking	Planned	Evaluator-feedback study in progress
Formal architectural evaluation (ATAM)	Planned	Final thesis cycle

Current evidence supports the feasibility of the approach and its architectural consistency with integrity and reliability expectations defined in ISO/IEC 25,010 [26]. A formal architectural evaluation using ATAM (Architecture Tradeoff Analysis Method) [27,28] is planned for the final thesis cycle in order to quantify tradeoffs and residual risks across the RM-ODP derived quality scenarios.

From an implementation status perspective, this validation cycle should be interpreted as Stage A complete and Stage B partial but operational. Stage B already produces running supervision services for repository evidence, while full multi-source closure across all stakeholder roles and event classes remains the next incremental objective.

To keep every claim proportional to demonstrated maturity, Table 5 classifies each capability of the method as implemented, partially implemented, or planned in the current cycle. Throughout this article, statements about operational behavior refer only to capabilities marked as implemented; partially implemented capabilities are operational strictly within the stated scope; planned capabilities are described as design intent.

## Limitations

The method presented in this work is designed to be generalizable to Project-Based Learning environments that produce observable digital traces of student activity. Nevertheless, several limitations must be acknowledged.

First, the method assumes the presence of digital platforms capable of generating structured event streams. Learning environments that rely primarily on offline activities, informal collaboration, or tools that do not expose event logs may not provide sufficient evidence for the Digital Twin to operate effectively. In such contexts, the observability layer becomes limited and the derived indicators may be incomplete.

Second, the current implementation cycle focuses primarily on repository-based evidence generated by version-control systems. While this source provides rich information about development cadence, artifact evolution, and collaboration patterns, it represents only one dimension of the broader PBL ecosystem. Other evidence channels such as task management systems, learning management platforms, mentoring interactions, and classroom activities are not yet fully integrated in the current cycle. As a result, the present instantiation should be interpreted as a partial implementation of the full method.

Third, the method depends on correct event interpretation and consistent data contracts. If connectors produce inconsistent data, if event semantics differ across platforms, or if institutional identifiers are not properly maintained, the normalization and correlation stages may produce misleading indicators. Institutions adopting the method must therefore ensure consistent data governance and stable integration interfaces.

Fourth, the architecture assumes that event processing within active sprint windows is technically feasible within the institutional infrastructure. Environments with strict infrastructure constraints, limited access to integration APIs, or restrictive data governance policies may experience delays or reduced event coverage, which can limit the responsiveness of the Digital Twin feedback cycle.

Finally, the method is intended to support supervision rather than to automate pedagogical decisions. Indicators and alerts generated by the Digital Twin highlight patterns and potential risks, but interpretation and intervention remain the responsibility of human stakeholders. The effectiveness of the approach therefore depends on the ability of instructors and coordinators to interpret the signals and incorporate them into their supervision practices.

These limitations do not invalidate the method but instead define the operational conditions under which it can be effectively applied. Future cycles of the research aim to expand multi-source evidence integration, strengthen governance mechanisms for event contracts, and evaluate the architecture across additional institutional contexts.

## Ethics statements

This study describes a methodological architecture and does not involve experiments with human participants, animals, or data collected from social media platforms. The quantitative evidence reported in the Method validation section derives from version-control repositories produced by one institutional team. All records were pseudonymized at source (truncated SHA-256 identifiers) before any processing, an automated scan verified the absence of cleartext personal identifiers in the analyzed corpus, and only team-level aggregates and pseudonymous indicators are reported, in accordance with institutional data-protection procedures under the Brazilian General Data Protection Law (LGPD). All language-model processing was executed on local institutional infrastructure; no data were transmitted to external services.

Any future empirical application of the method should comply with institutional ethics policies and data protection regulations, including informed consent procedures and anonymization when applicable.

## Related research article

None.

## For a published article

None.

## Supplementary material *and/or* additional information [OPTIONAL]

Not applicable.

## CRediT authorship contribution statement

**Afonso Cesar Lelis Brandão:** Conceptualization, Methodology, Software, Investigation, Writing – original draft, Visualization. **Reginaldo Arakaki:** Conceptualization, Methodology, Validation, Writing – review & editing. **Leandro Augusto da Silva:** Supervision, Project administration, Writing – review & editing.

## Declaration of competing interest

The authors declare that they have no known competing financial interests or personal relationships that could have appeared to influence the work reported in this paper.

## Data Availability

No data was used for the research described in the article.
